# Antibiotikaprophylaxe bei transrektaler Prostatabiopsie: Im Kontext von Fluorchinolon-Indikationsrücknahme und Antibiotic Stewardship

**DOI:** 10.1007/s00120-021-01618-1

**Published:** 2021-08-18

**Authors:** Kathrin Rothe, Christiane Querbach, Dirk H. Busch, Jürgen E. Gschwend, Katharina Hauner

**Affiliations:** 1 Institut für Medizinische Mikrobiologie, Immunologie und Hygiene, Technische Universität München Fakultät für Medizin, München, Deutschland; 2 Krankenhausapotheke Klinikum rechts der Isar, Technische Universität München Fakultät für Medizin, München, Deutschland; 3 Urologische Klinik und Poliklinik, Klinikum rechts der Isar, Technische Universität München Fakultät für Medizin, Ismaninger Str. 22, 81675 München, Deutschland

**Keywords:** Fluorchinolon-PAP, Infektiöse Komplikationen, Klinische Praxis der PAP, Gezielte Prophylaxe, Antibiotikaresistenz, Clinical practice of PAP, Infectious complications, Antimicrobial resistance, Targeted PAPs, Fluoroquinolone-PAP

## Abstract

**Hintergrund:**

Die transrektale Prostatastanzbiopsie (TRPB) gehört zu den häufigsten interventionell-urologischen Eingriffen in Deutschland. Es wird leitliniengerecht eine kurzeitige periprozedurale Antibiotikaprophylaxe (PAP) empfohlen. Die Indikationsrücknahme von Fluorchinolonen als PAP-Substanz durch das Bundesinstitut für Arzneimittel und Medizinprodukte macht die Verwendung alternativer Substanzen notwendig.

**Ziele:**

Im Rahmen der Studie wurde die klinische Praxis der PAP bei TRPB mit Fokus auf infektiöse Komplikationen im Vergleich zwischen Fluorchinolon- und Cotrimoxazol-PAP evaluiert.

**Methodik:**

Es handelt sich um eine retrospektive monozentrische Auswertung klinischer Routinedaten von Patienten mit TRPB zwischen 03.01.2019 und 28.01.2021.

**Ergebnisse:**

Es wurden 508 erwachsene männliche Patienten eingeschlossen, das mediane Alter betrug 68 Jahre. 55,9 % erhielten eine Cotrimoxazol-PAP, 40,0 % eine Fluorchinolon-PAP. Insgesamt traten in 5,5 % postinterventionelle Komplikationen auf, davon 50,0 % infektiöse Komplikationen. Der Vergleich von Cotrimoxazol- und Fluorchinolon-PAP ergab keinen Unterschied der Komplikationsraten. Bei aufgrund von Komplikationen durchgeführten mikrobiologischen Urinuntersuchungen zeigten sich Erregernachweise mit Resistenz gegenüber der zuvor eingesetzten PAP im Sinne einer Selektion.

**Schlussfolgerung:**

Eine Cotrimoxazol-PAP für TRPB ist verglichen mit dem bisherigen Standard einer Fluorchinolon-PAP nicht mit vermehrt infektiösen Komplikationen assoziiert. Die präinterventionelle Analyse von Keimspektrum und Resistenz ermöglicht den Einsatz einer gezielten Prophylaxe und kann somit Komplikationen reduzieren.

## Abstract (en)

**Background:**

Transrectal prostate biopsy (TRPB) is the gold standard for prostate cancer diagnosis and among the most common urological interventions. Short-term antibiotic prophylaxis (PAP) is recommended for TRPB. Fluoroquinolone-PAP as standard of care needs to be revaluated due to the restrictions on the use of fluoroquinolone antibiotics by the German Federal Institute for Drugs and Medical Devices.

**Objectives:**

The aim of the study was to analyze clinical practice of PAP for TRPB with focus on infectious complications and potential differences between fluoroquinolone-PAP and cotrimoxazole-PAP.

**Methods:**

We performed a retrospective monocentric study of clinical and microbiological characteristics of patients with TRPB between 3 January 2019 and 28 January 2021.

**Results:**

A total of 508 men were included; median age was 68 years. In all, 55.9% of our cohort received cotrimoxazole-PAP and 40.0% fluoroquinolone-PAP. Postinterventional complications occurred in 5.5%, of those 50.0% were infectious complications. Complication rate did not differ between patients with fluoroquinolone-PAP and cotrimoxazole-PAP. Urinary cultures in case of postinterventional complications yielded pathogens with antimicrobial resistance against the used PAP substance indicating selection of resistant bacteria.

**Conclusion:**

Cotrimoxazole-PAP for TRPB is not associated with an increase of infectious complications compared to fluoroquinolone-PAP. Cultures obtained prior to TRPB to identify antimicrobial resistance facilitate targeted PAP and therefore can reduce complications.

## Hintergrund

Die transrektale Prostatastanzbiopsie gehört zu den häufigsten interventionell-urologischen Eingriffen in Deutschland. Hier wird leitliniengerecht eine kurzeitige periprozedurale Antibiotikaprophylaxe empfohlen. Die Indikationsrücknahme von Fluorchinolonen durch das Bundesinstitut für Arzneimittel und Medizinprodukte macht die Verwendung alternativer Prophylaxesubstanzen hierfür notwendig, wobei hier bislang kein Konsens über die Substanzwahl besteht. Im Rahmen dieser Studie soll der Einsatz von Cotrimoxazol für diese Indikation evaluiert werden.

Das Prostatakarzinom ist die häufigste Tumorerkrankung des Mannes. Zur Abklärung eines suspekten Befunds werden Stanzzylinder zur histologischen Aufarbeitung mittels Prostatastanzbiopsie gewonnen. Da die perineale Biopsie in der Regel durch den Einsatz von Templates technisch aufwändiger ist und traditionell in Kurznarkose durchgeführt wird, findet in der Praxis bislang meist die transrektale Biopsie (TRPB) Anwendung [15, 26]. TRPB gehören mit ca. 150.000 Biopsien pro Jahr in Deutschland zu den häufigsten interventionell-urologischen Eingriffen [4, 5, 9]. Die TRPB geht mit einem Risiko für Hämaturie und Infektionen einher. Periprozedural wird leitliniengerecht eine kurzeitige Antibiotikaprophylaxe (PAP) durchgeführt, was infektiöse Komplikationen reduziert. Es existiert kein Konsens, welche antibiotische Substanz und Dauer als optimal anzusehen sind. Bislang wurden aufgrund Bioverfügbarkeit und Gewebegängigkeit vorrangig Fluorchinolone (FQ) eingesetzt. Parallel zu den zunehmenden Resistenzen der gramnegativen Darmflora gegenüber FQ zeigt sich ein über die letzten 10–15 Jahre ansteigender Trend postinterventioneller Infektionsraten nach TRPB. FQ-Resistenz wird als Risikofaktor für postinterventionelle Infektionen nach TRPB angesehen, so wie generell Unwirksamkeit der verabreichten PAP [2, 8, 16, 22, 24, 28]. Inzwischen sind FQ zudem aufgrund des Nebenwirkungsprofils als kritisch anzusehen [11, 19]. Die EU-Kommission hat im Zuge einer Nutzen-Risiko-Bewertung im März 2019 die Indikation für FQ als PAP für Eingriffe am Urogenitaltrakt wie TRPB aufgrund des Risikos für das Auftreten lang anhaltender und beeinträchtigender Nebenwirkungen im Bereich Muskeln, Gelenke und Nervensystem zurückgezogen (Durchführungsbeschluss C[2019]2050) und es besteht seit 30.04.2019 eine nationale Indikationsrücknahme (GESCHZ75.02-3822-V-18245-8001/19) für die Prophylaxe für Eingriffen am Urogenitaltrakt durch das Bundesinstitut für Arzneimittel und Medizinprodukte (BfArM).

Als Reaktion auf die Indikationsrücknahme wurde die hausinterne Leitlinie zur PAP bei TRPB in interdisziplinärer Zusammenarbeit mit dem Antibiotic-Stewardship(ABS)-Team überarbeitet und nach interner Evaluation und Konsensusbildung Ende 2019 fertiggestellt. Die Möglichkeit alternativer oraler PAP-Substanzen wurde intern diskutiert. So wurde neben Fosfomycin [7, 10, 18] auch eine Kombination aus Pivmecillinam und Amoxicillin/Clavulansäure [1] erörtert. Fosfomycin-PAP ist insbesondere für Erreger mit niedriger minimaler Hemmkonzentration empfohlen [21] und Fosfomycin ist ein wertvolles Breitspektrumreserveantibiotikum, dessen Resistenzentwicklung mit geringen therapeutischen Spiegeln und vermehrtem Einsatz assoziiert ist, sodass ein Selektionsdruck bei vermehrt Nicht-Harnwegsinfektions(HWI)-Indikationen zu befürchten ist [13, 23]. Daher wurde zunächst auch auf Basis publizierter Leitlinien, und Daten sowie der internen Resistenzlage [3, 14, 30] das empfohlene Antibiotikaregime konsensuell von Ciprofloxacin zu Cotrimoxazol (SXT) angepasst. Dieses Vorgehen sollte im Rahmen der aktuellen Studie evaluiert werden. Standardmäßig wurde nach Implementierung der neuen Leitlinie seit Januar 2020 SXT 960 mg p.o. 3‑mal periinterventionell verabreicht: Am Vorabend der TRPB sowie am Morgen (2–4 h vor dem Eingriff) und Abend des Interventionstages. Im Rahmen dieser Studie soll die Praxis der PAP bei TRPB mit besonderem Augenmerk auf postinterventionelle infektiöse Komplikationen im Vergleich des Zeitraums vor und nach Umstellung des internen PAP-Standards evaluiert werden.

## Methodik

Retrospektive monozentrische Studie auf Basis klinischer Routinedaten von 508 Patienten mit TRPB an einem Universitätsklinikum zwischen 03.01.2019 und 28.01.2021. Gemäß dem internen Standard wurde präinternvetionell keine lokale intrarektale Povidon-Iod-Desinfektion durchgeführt. Patienten wurden für 30 Tage postinterventionell nachverfolgt. Demographische Daten, Komorbiditäten, klinische Verläufe, mikrobiologische Diagnostik, therapeutisches Management und antibiotische Substanzen wurden anhand elektronischer Patientenakten und dem Laborinformationssystem des Instituts für medizinische Mikrobiologie erhoben. Die Variablen wurden vorab definiert, durch das Studienteam erhoben und ausgewertet. Postinterventionelle HWI wurden als komplizierte untere HWI gewertet, sofern es klinisch oder sonographisch keinen Anhalt für eine Pyelonephritis gab. Die Zustimmung der Ethikkommission der Technischen Universität München zur Durchführung der Studie liegt vor (Votum 395/20 S).

Qualitative Daten sind als Median und Spannweite angegeben, quantitative Daten als absolute und relative Häufigkeiten. Statistische Gruppenvergleiche erfolgten mittels χ^2^-Test bzw. Mann-Whitney-U-Test je mit 5 %-Signifikanzlevel. Die Auswertung erfolgte mittels Microsoft Excel 2016 (Microsoft Corp, Redmond, WA, USA) und IBM SPSS Statistics Version 26 (IBM Corp, Armonk, NY, USA).

## Ergebnisse

Es wurden 508 erwachsene männliche Patienten eingeschlossen, das mediane Alter betrug 68 Jahre. 55,9 % erhielten eine SXT-PAP und 40,0 % eine FQ-PAP. Im zeitlichen Verlauf erfolgte zunehmend (jedoch nicht für alle Patienten der Kohorte) präinterventionell ein rektales Screening auf multiresistente gramnegative Keime (MRGN-Screening) und eine Urinkultur. Das MRGN-Screening war kein definitiver interner Standard, lag nur für 50,0 % der Kohorte vor und führte nicht konsequent zu einer adäquaten Anpassung der PAP-Substanz, sodass dies nicht als „targeted prophylaxis“ gewertet werden konnte. Es zeigte sich eine niedrige 3MRGN-Rate (5,3 %). In keinem der MRGN-Fälle entwickelte sich bei testgerechter PAP mit einem Carbapenem (57,1 %) bzw. SXT eine postinterventionelle Komplikation. Die präinterventionellen Urinkulturen zeigten sich überwiegend steril (79,0 %), die häufigsten nachgewiesenen Erreger waren *Enterobacterales *und* Enterobacter faecalis*. Interessanterweise waren Patienten mit Enterokokkennachweis älter (Median 72 Jahre). Insgesamt traten bei 5,5 % der TRPB postinterventionelle Komplikationen auf. Hiervon waren 50,0 % infektiöse Komplikationen, hiervon der überwiegende Anteil untere HWI. In 82,1 % aller Komplikationen war ein transurethaler Katheter erforderlich, in 53,6 % wurde eine antibiotische Therapie gestartet und in 85,7 % eine Urinkultur entnommen. Diese Kulturen zeigten sich in 50,0 % der Fälle positiv mit einem uropathogenen Erreger, wobei es sich hierbei um 75,0 % SXT-resistente und 59,3 % FQ-resistente Erreger handelte. Eine Urosepsis (konkordanter Erregernachweis in Urin und Blutkultur) als Komplikation war mit drei Fällen sehr selten. Bei Urosepsis fand sich innerhalb von 1–5 Tagen: *Escherichia coli* (FQ resistent, SXT sensibel) nach FQ-PAP, *Escherichia coli* (FQ sensibel, SXT resistent) nach SXT-PAP und *Pseudomonas aeruginosa* (FQ resistent, SXT resistent) nach SXT-PAP auf (Tab. 1).

**Table 1:** 

Relevante Kohortencharakteristika	Absolute/relative Häufigkeit (bzw. Median)
*Alter in Jahren, Median (Spannweite)*	*68 (24–90)*
*PAP-Substanz*	
SXT	284/508 (55,9 %)
Ciprofloxacin	188/508 (37,0 %)
Levofloxacin	15/508 (3,0 %)
Meropenem	12/508 (2,4 %)
Amoxicillin/Clavulansäure	9/508 (1,8 %)
*PAP-Dauer in Tagen, Median (Spannweite)*	*3,5 (2–6)*
*Vorliegende Komorbidität*^*a*^	*166/479 (34,7* *%)*
*3MRGN rektal (E. coli n* *=* *12, K. pneumoniae n* *=* *1, C. freundii n* *=* *1) vor TRPB*	*14/266 (5,3* *%)*
*Positive Urinkultur vor TRPB*^*b*^	*83/395 (21,0* *%)*
*Komplikation nach TRPB*	*28/508 (5,5* *%)*
Fieber > 38,5 °C	11/28 (39,3 %)
Sepsis	5/28 (17,9 %)
Dysurie	21/28 (75,0 %)
Blutung	10/28 (35,7 %)
Harnverhalt	17/28 (60,7 %)
Infektion	14/28 (50,0 %)
*Hospitalisierung*	*16/28 (57,1* *%)*
*Antibiotische Therapie*^*c*^	*15/28 (53,6* *%)*
*Positive Urinkultur 1–15* *d nach TRPB (E. coli n* *=* *11, P. aeruginosa n* *=* *1)*	*12/24 (50,0* *%)*
3MRGN	2/12 (16,7 %)
FQ-PAP vor Komplikation	5/12 (41,7 %)
SXT-PAP vor Komplikation	7/12 (58,3 %)
*Blutstrominfektion (E. coli n* *=* *2, P. aeruginosa n* *=* *1) 0–5* *d nach TRPB*	*3/7 (42,9* *%)*
*Biopsie*	
Histologischer Nachweis Prostatakarzinom	342/508 (67,3 %)
Anzahl Biopsiezylinder, Median (Spannweite)	15 (3–36)

*TRPB* transrektale Prostatastanzbiopsie, *PAP* periprozedurale Antibiotikaprophylaxe, *SXT* Cotrimoxazol, *MRGN* multiresistente gramnegative Keime, *FQ* Fluorchinolone

^a^Adipositas, arterielle Hypertension, Diabetes mellitus, koronare Herzkrankheit, chronische Niereninsuffizienz, systemische Tumorerkrankung, Immunsuppression, Lebererkrankung

^b^*E. faecalis n* = 41, *P. aeruginosa n* = 5, *S. aureus n* = 1, *S. maltophilia n* = 1, *C. koseri n* = 5, *E. coli n* = 23, *K. aerogenes n* = 1, *K. pneumoniae n* = 4, *M. morganii n* = 6, *K. oxytoca n* = 4, *P. mirabilis n* =6

^c^Piperacillin/Tazobactam *n* = 5, Ciprofloxacin *n* = 6, Meropenem *n* = 3, Pivmecillinam *n* =1

Der Vergleich von SXT-PAP mit FQ-PAP ergab keinen signifikanten Unterschied der Komplikationsraten nach TRPB. Die SXT-Gabe wurde stringenter auf 2 Tage limitiert, während die FQ-Gabe im Mittel 5 Tage betrug. Patienten in der FQ-Gruppe litten häufiger an Vorerkrankungen, insbesondere Diabetes mellitus. Uropathogene Erregernachweise im präinterventionellen Urinkulturscreening waren häufiger in der FQ-Gruppe, wobei hier vermehrt *Enterobacter faecalis *nachweisbar war. Da dieser als SXT-resistent anzusehen ist und den behandelnden Ärzten der Einsatz von Levofloxacin bei *Enterobacter-faecalis-*Nachweise offen stand, ist die Häufung von *Enterobacter faecalis *in der FQ-Gruppe und die längere Dauer der FQ-Gabe nachvollziehbar. Dahingegen waren in der SXT-Gruppe in präinterventionellen Urinen überwiegend *Enterobacterales* in Reinkultur nachweisbar. Im klinischen Management der postinterventionellen Komplikationen (u. a. Dauer des Krankenhausaufenthalts und transurethrale Katheteranlage) waren die beiden Gruppen vergleichbar. Die Gruppen unterschieden sich nicht hinsichtlich der Zeitspanne zwischen Biopsie und Auftreten der Komplikation. Bei aufgrund von Komplikationen durchgeführten mikrobiologischen Urinuntersuchungen zeigte sich eine Tendenz hin zu Erregernachweisen mit Resistenz gegenüber der zuvor eingesetzten PAP im Sinne einer Selektion (Tab. 2).

**Table 2:** 

Charakteristika [absolute/relative Häufigkeit]^a^	FQ-PAP	SXT-PAP	Gesamtkohorte	*p*-Wert^b^
*Alter in Jahren, Median (Spannweite); n* *=* *487*	*69 (24–87)*	*67 (46–90)*	*68 (24–90)*	*0,50*
*PAP-Dauer in Tagen, Median (Spannweite); n* *=* *487*	*5 (5–5)*	*2 (2–2)*	*4 (2–5)*	0,00
*Vorliegende Komorbidität*^*c*^	*96/193 (49,7* *%)*	*64/266 (24,1* *%)*	*160/459 (34,9* *%)*	0,00
Adipositas	21/193 (10,9 %)	23/266 (8,6 %)	44/459 (9,6 %)	0,42
Diabetes mellitus	17/193 (8,8 %)	11/266 (4,1 %)	28/459 (6,1 %)	0,04
*MRGN-Screening vor TRPB positiv*	*0/31 (0,0* *%)*	*6/221 (2,7* *%)*	*6/252 (2,4* *%)*	*0,35*
*Positive Urinkultur vor TRPB*	*44/133 (33,1* *%)*	*25/241 (10,4* *%)*	*69/374 (18,4* *%)*	0,00
*Enterobacterales*	13/44 (29,5 %)	16/25 (64,0 %)	29/69 (42,0 %)	0,01
*E. faecalis* & *Enterobacterales*	7/44 (15,9 %)	1/25 (4,0 %)	8/69 (11,6 %)	0,14
*E. faecalis*	21/44 (47,7 %)	3/25 (12,0 %)	24/69 (34,8 %)	0,00
*P. aeruginosa*	3/44 (6,8 %)	1/25 (4,0 %)	4/69 (5,8 %)	0,63
FQ-Resistenz	2/44 (4,5 %)	2/25 (8,0 %)	4/69 (5,8 %)	0,56
SXT-Resistenz	33/44 (75,0 %)	4/25 (16,0 %)	37/69 (53,6 %)	0,00
*Komplikation nach TRPB*	*13/203 (6,4* *%)*	*14/284 (4,9* *%)*	*27/487 (5,5* *%)*	*0,48*
Fieber > 38,5 °C	4/13 (30,8 %)	7/14 (50,0 %)	11/27 (40,7 %)	0,31
Sepsis	3/13 (23,1 %)	2/14 (14,3 %)	5/27 (18,5 %)	0,56
Dysurie	11/13 (84,6 %)	10/14 (71,4 %)	21/27 (77,8 %)	0,41
Blutung	4/13 (30,8 %)	5/14 (35,7 %)	9/27 (33,3 %)	0,79
Harnverhalt	10/13 (76,9 %)	6/14 (42,9 %)	16/27 (59,3 %)	0,07
Infektion	6/13 (46,2 %)	8/14 (57,1 %)	14/27 (51,9 %)	0,57
*Hospitalisierung*	*7/13 (53,8* *%)*	*8/14 (57,1* *%)*	*15/27 (55,6* *%)*	*0,86*
*Antibiotische Therapie*	*8/13 (61,5* *%)*	*7/14 (50,0* *%)*	*15/27 (56,6* *%)*	*0,55*
*Positive Urinkultur*	*5/12 (41,7* *%)*	*7/12 (58,3* *%)*	*12/24 (50,0* *%)*	*0,41*
FQ-Resistenz	5/5 (100,0 %)	2/7 (28,6 %)	7/12 (58,3 %)	0,01
SXT-Resistenz	2/5 (40,0 %)	7/7 (100,0 %)	9/12 (75,0 %)	0,02
*Blutstrominfektion*	*1/3 (33,3* *%)*	*2/4 (50,0* *%)*	*3/7 (42,9* *%)*	*0,66*

*TRPB* transrektale Prostatastanzbiopsie, *PAP* periprozedurale Antibiotikaprophylaxe, *SXT* Cotrimoxazol, *MRGN* multiresistente gramnegative Keime, *FQ* Fluorchinolone

^a^Aufgrund des retrospektiven Studienansatzes waren nicht alle Parameter für alle Patienten verfügbar, was unterschiedliche Gruppengrößen zur Folge hat (durch „N“ gekennzeichnet)

^b^Gruppenvergleich χ^2^- bzw. Mann-Whitney-U-Test

^c^s. Tab. 1

Um Faktoren zu ermitteln, welche mit infektiösen Komplikationen nach TRPB assoziiert sind, wurden Patientencharakteristika bei Fällen mit bzw. ohne Komplikation verglichen. Erneut zeigte sich kein Einfluss von PAP-Dauer- und Substanz. Keine der erfassten Komorbiditäten hatte einen Einfluss auf infektiöse komplikative Verläufe. In unserer Kohorte war das Vorhandensein von 3MRGN im Screeningabstrich ebenso wenig ein Prädiktor für Komplikationen wie präintervenionelle positive Urinkulturen. Dies zeigt, dass die präinterventionelle Analyse von Keimspektrum und Resistenzen eine erfolgreiche gezielte PAP ermöglicht und somit Komplikationen reduziert (Tab. 3).

**Table 3:** 

Charakteristika (absolute/relative Häufigkeit)^a^	Keine Komplikation	Komplikation	Gesamtkohorte	*p*-Wert^b^
*Alter in Jahren, Median (Spannweite); n* *=* *508*	*68 (24–90)*	*69 (49–78)*	*68 (24–90)*	*0,94*
*PAP-Dauer in Tagen, Median (Spannweite); n* *=* *508*	*4 (2–5)*	*2 (2–5)*	*4 (2–5)*	*0,99*
*PAP-Substanz*				*0,73*
FQ	197/494 (39,9 %)	6/14 (42,9 %)	203/508 (40,0 %)	–
SXT	276/494 (55,9 %)	8/14 (57,1 %)	284/508 (55,9 %)	–
Andere	21/4894 (4,3 %)	0/14 (0,0 %)	21/508 (4,1 %)	–
*Vorliegende Komorbidität*^*c*^	*159/465 (34,2* *%)*	*7/14 (50,0* *%)*	*166/479 (34,7* *%)*	*0,22*
*MRGN-Screening vor TRPB positiv*	*14/259 (5,4* *%)*	*0/7 (0,0* *%)*	*14/266 (5,3* *%)*	*0,53*
*Positive Urinkultur vor TRPB*	*81/382 (21,2* *%)*	*2/13 (15,4* *%)*	*83/395 (21,0* *%)*	*0,61*
3MRGN	6/81 (7,4 %)	0/2 (0,0 %)	6/83 (7,2 %)	0,69
*Enterobacterales*	33/81 (40,7 %)	0/2 (0,0 %)	33/83 (39,8 %)	0,25
*E. faecalis* & *Enterobacterales*	10/81 (12,3 %)	0/2 (0,0 %)	10/83 (12,0 %)	0,60
*E. faecalis*	30/81 (37,0 %)	1 /2 (50,0 %)	31/81 (37,3 %)	0,71
*P. aeruginosa*	5/81 (6,2 %)	0/2 (0,0 %)	5/83 (6,0 %)	0,72
FQ-Resistenz	10/81 (12,3 %)	0/2 (0,0 %)	10/83 (12,0 %)	0,60
SXT-Resistenz	50/81 (61,7 %)	1/2 (50,0 %)	51/83 (61,4 %)	0,74

*TRPB* transrektale Prostatastanzbiopsie, *PAP* periprozedurale Antibiotikaprophylaxe, *SXT* Cotrimoxazol, *MRGN* multiresistente gramnegative Keime, *FQ* Fluorchinolone

^a^s. Tab. 2

^b^s. Tab. 2

^c^s. Tab. 1

## Diskussion und Limitationen

Die TRPB ist der Goldstandard zur Diagnosesicherung des Prostatakarzinoms, postinterventionelle Infektionsraten liegen bei 0,1–7,0 % [17]. In dieser Studie traten bei 5,5 % der TRPB Komplikationen auf, davon 50 % infektiöse Komplikationen. Der Vergleich von SXT-PAP mit FQ-PAP ergab keinen Unterschied der Komplikationsraten. Der Einsatz von SXT als empirische PAP für TRPB kann somit als adäquate Option angesehen werden.

Bei infektiösen Komplikationen nach TRPB scheinen FQ-resistente Erreger eine besondere Rolle zu spielen und das postintervenionelle Infektionsrisiko zu erhöhen. Risikofaktoren und antimikrobielle Resistenz sollten im Vorfeld der TRPB kontrolliert werden [6, 17]. Um auf zunehmende Resistenzen zu reagieren, wird teilweise der Einsatz von Reserveantibiotika als PAP empfohlen („augmented prophylaxis“). Die ist aufgrund potenzieller Resistenzinduktion und nach ABS-Grundsätzen, welche eine Einsparung von Reserveantibiotika fordern, kritisch anzusehen [8].

Dem gegenüber stehen Screening-basierte PAP(„targeted prophylaxis“)-Strategien als vielversprechender Ansatz, um eine testgerechte periprozedurale PAP durchzuführen und Reserveantibiotika einzusparen. Hier werden präinterventionell Rektalabstriche entnommen und auf Resistenzen gegenüber ausgewählten PAP-Substanzen gescreent [8, 12, 25, 27–29, 31]. Dieses Screening relevanter PAP-Substanzen ist im klinischen Alltag nicht etabliert, da kommerzielle Selektivmedien fehlen. Eine verfügbare kommerzielle Methode existiert nur für das Screening auf FQ-Resistenz [8]. Durch die Vielzahl gramnegativer Bakterien der Darmflora ist es nicht möglich, routinemäßig einzelne Resistenztestungen für alle vorhandenen Erreger durchzuführen. Durch das übliche MRGN-Screening erfolgt jedoch keine Überprüfung von insbesondere für die PAP relevanten Substanzen.

Der gezielte PAP-Einsatz kann die Infektkomplikationsrate senken [6, 20, 31]. Rektalabstriche vor TRPB ermöglichen eine resistogrammgerechte PAP. Die Kenntnis über die lokale Resistenzlage kann zudem in lokale PAP-Strategien im Sinne von ABS-Prinzipien umgesetzt werden. Außerdem ist bei Patienten mit Symptomen im unteren Harntrakt eine Urinkultur mit testgerechter Therapie vor TRPB zu empfehlen [17]. Daher wird an unserem Zentrum zunehmend vor TRPB ein MRGN-Screening und eine Urinkultur durchgeführt. In unserer Kohorte war das Vorhandensein von 3MRGN im Screeningabstrich ebenso wenig ein Prädiktor für Komplikationen wie präintervenionelle positive Urinkulturen. Da diese Befunde den Einsatz der PAP-Substanz beeinflussen, zeigt dies, dass die präinterventionelle Analyse von Keimspektrum und Resistenz eine gute gezielte PAP ermöglicht und somit Komplikationen reduziert. Eine Umstellung auf die transperineale Prostatastanzbiopsie und damit eine vollständige Vermeidung der Einbringung von Rektalflora zur Reduktion von Infektkomplikationen ist sicherlich auch im Rahmen des ABS-Managements zu diskutieren [15, 26].

## Ausblick

Während in unserer Kohorte durch testgerechte PAP basierend auf MRGN-Screening und präintervenioneller Urinkultur eine sehr niedrige Infektkomplikationsrate vorlag, zeigte sich bei aufgrund von Komplikationen durchgeführten Urinkulturen eine Tendenz hin zu Erregernachweisen mit Resistenz gegenüber der zuvor eingesetzten PAP. Dies bestätigt, dass durch präinterventionelle Resistenzanalyse mittels gezielter PAP Komplikationen reduziert werden können. Für einen breiten kosteneffizienten Einsatz der gezielten PAP-Strategie und somit Reduktion des Einsatzes von Reserveantibiotika wäre eine Testung aller in diesem Zusammenhang relevanten antibiotischen Substanzen im klinischen Alltag mittels kommerzieller Selektivmedien wünschenswert.

## Fazit für die Praxis

- Der empirische Einsatz der SXT-PAP (Cotrimoxazol-periprozedurale Antibiotikaprophylaxe) für TRPB ist im Vergleich zu FQ-PAP (Fluorchinolon-periprozedurale Antibiotikaprophylaxe) nicht mit vermehrt postinterventionellen infektiösen Komplikationen assoziiert und kann somit als Reaktion auf die Indikationsrücknahme von FQ in diesem Kontext als adäquate Alternative angesehen werden.
- Da eine Unwirksamkeit der verabreichten PAP als Risikofaktor für postinterventionelle Infektionen nach transrektaler Prostatastanzbiopsie (TRPB) anzusehen ist, wäre eine routinemäßige präinterventionelle Analyse von individuellem Keimspektrum und Resistenz hinsichtlich zunehmender weltweiter Antibiotikaresistenzen wünschenswert. Ein Screening-basierter testgerechter Einsatz der PAP kann Komplikationen reduzieren und den Einsatz von Reserveantibiotika als PAP reduzieren was unter ABS-Gesichtspunkten (Antibiotic Stewardship) anzustreben ist.
